# Supplementary material for: Piezo2 expressing nociceptors mediate mechanical sensitization in experimental osteoarthritis
Source: Nat Commun. 2023 Apr 29;14:2479. doi: 10.1038/s41467-023-38241-x (PMC10148822; doi:10.1038/s41467-023-38241-x)
Supplement: Supplementary file 1 — Supplementary Information [file 41467_2023_38241_MOESM1_ESM.pdf]

Supplementary Information for:

Piezo2 expressing nociceptors mediate mechanical sensitization in experimental osteoarthritis

Authors: Alia M. Obeidat[1]†, Matthew J. Wood[1]†, Natalie S. Adamczyk[1], Shingo Ishihara[1], Jun Li[1], Lai Wang[1], Dongjun Ren[2], David A. Bennett[3], Richard J. Miller[2], Anne-Marie Malfait[1], Rachel E. Miller[1]*

Affiliations:

[1]Department of Internal Medicine, Division of Rheumatology, Rush University Medical Center; Chicago, USA.

[2]Department of Pharmacology, Northwestern University; Chicago, USA.

[3]Rush Alzheimer's Disease Center and Department of Neurological Sciences, Rush University Medical Center; Chicago, USA.

†These authors contributed equally to this work.

*Corresponding author. Email: Rachel_Miller@rush.edu.

Supplementary Information file

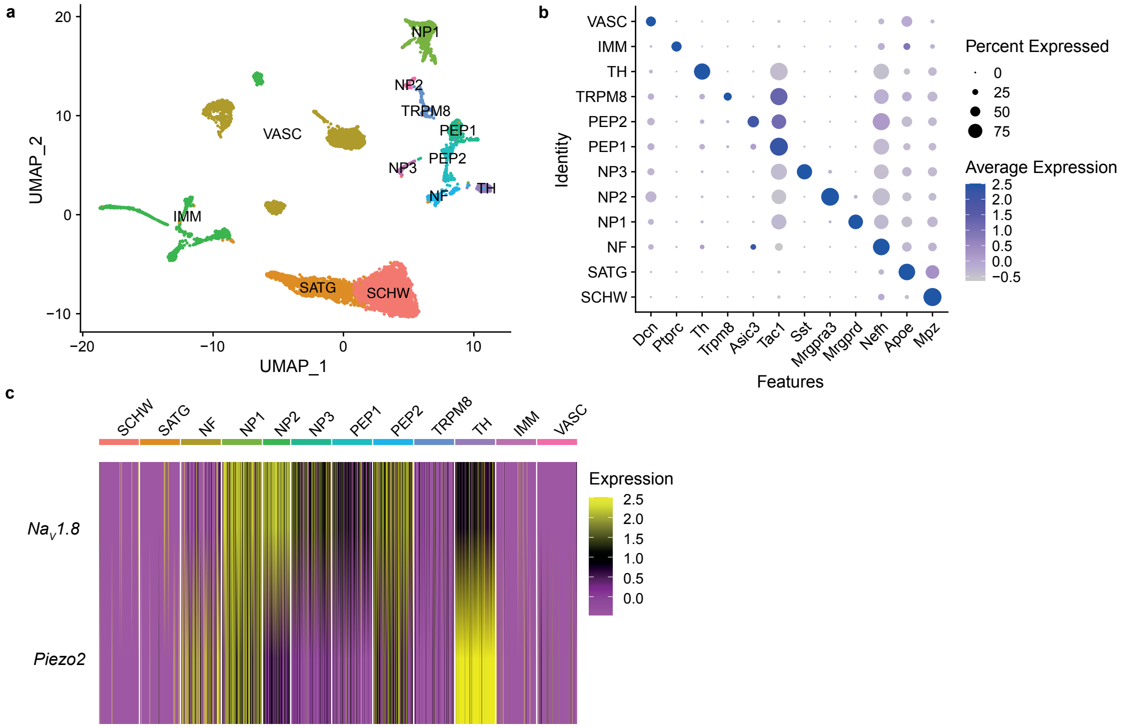

d

Gene	Cluster	p_val	avg_log2FC	pct.1	pct.2	p_val_adj
Piezo2	NF	2.58E-51	1.624	0.635	0.199	5.22E-47
Piezo2	NP1	0	1.557	0.898	0.152	0
Piezo2	PEP2	2.94E-88	0.759	0.829	0.193	5.94E-84
Piezo2	TH	2.55E-165	2.760	1	0.195	5.15E-161

e

Cluster Name	SCHW	SATG	NF	NP1	NP2	NP3	PEP1	PEP2	TRPM8	TH	IMM	VASC
# Cells	2801	1483	178	656	68	110	282	205	154	139	1037	1642

Supplemental Figure 1. Additional single cell RNAseq information associated with Figure 1. **a** UMAP plot of 8,755 L3-L5 dorsal root ganglia cells isolated from 18-week old male naïve C57BL/6 mice. Cells were clustered and labeled based on previously published datasets (*21, 80*). 'SCHW' = Schwann cells; 'SATG' = satellite glia; 'NF' = neurofilament; 'NP' = non-peptidergic nociceptors; 'PEP' = peptidergic nociceptors; 'TRPM8' = transient receptor potential melastatin 8; 'TH' = tyrosine hydroxylase containing; 'IMM' = immune cells; 'VASC' = vascular cells; clusters follow annotation strategy as in (Zeisel A*, et al.* Molecular Architecture of the Mouse Nervous System. *Cell* **174**, 999-1014 e1022 (2018); Usoskin D*, et al.* Unbiased classification of sensory neuron types by large-scale single-cell RNA sequencing. *Nat Neurosci* **18**, 145-153 (2015)). **b** Example marker genes used to identify each cluster. **c** Heatmap displaying expression of *$Na_V1.8$* and *Piezo2* on a cell-by-cell basis. **d** FindAllMarkers command in Seurat used to identify clusters with upregulated *Piezo2* expression (Wilcoxon rank-sum test option). NF = *$Na_V1.8$-* neurons; NP1, PEP2, TH = *$Na_V1.8$+* neurons. **e** Number of cells in each cluster.

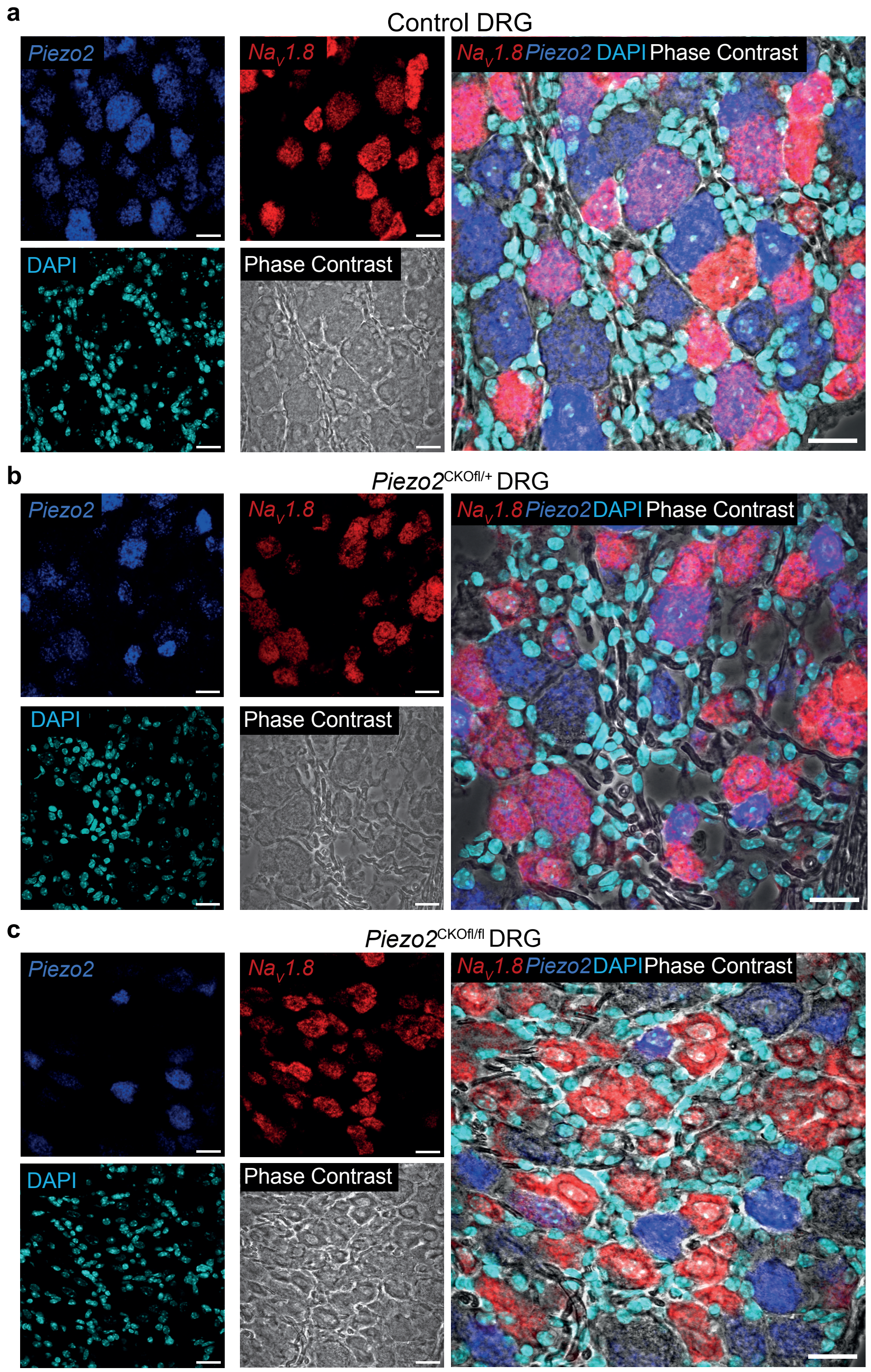

Supplemental Figure 2. Additional RNAscope pictures of mouse dorsal root ganglia (DRG) from same group of mice used in Figure 1. Pictures shown are examples from different mice than in Figure 1. **a** control, **b** *Piezo2*$^{\mathrm{CKOfl/+}}$ or **c** *Piezo2*$^{\mathrm{CKOfl/fl}}$ DRG with phase contrast and DAPI channels shown. Scale bar = 25 µm. n=4 no Cre controls; n=3 *Piezo2*$^{\mathrm{CKOfl/+}}$; n=5 *Piezo2*$^{\mathrm{CKOfl/fl}}$. For **a**, **b**, **c**, adjustments to individual colour channels on merged images was performed using brightness and contrast tools. Adjustments were made applied to the entire image and have been applied across all images and controls.

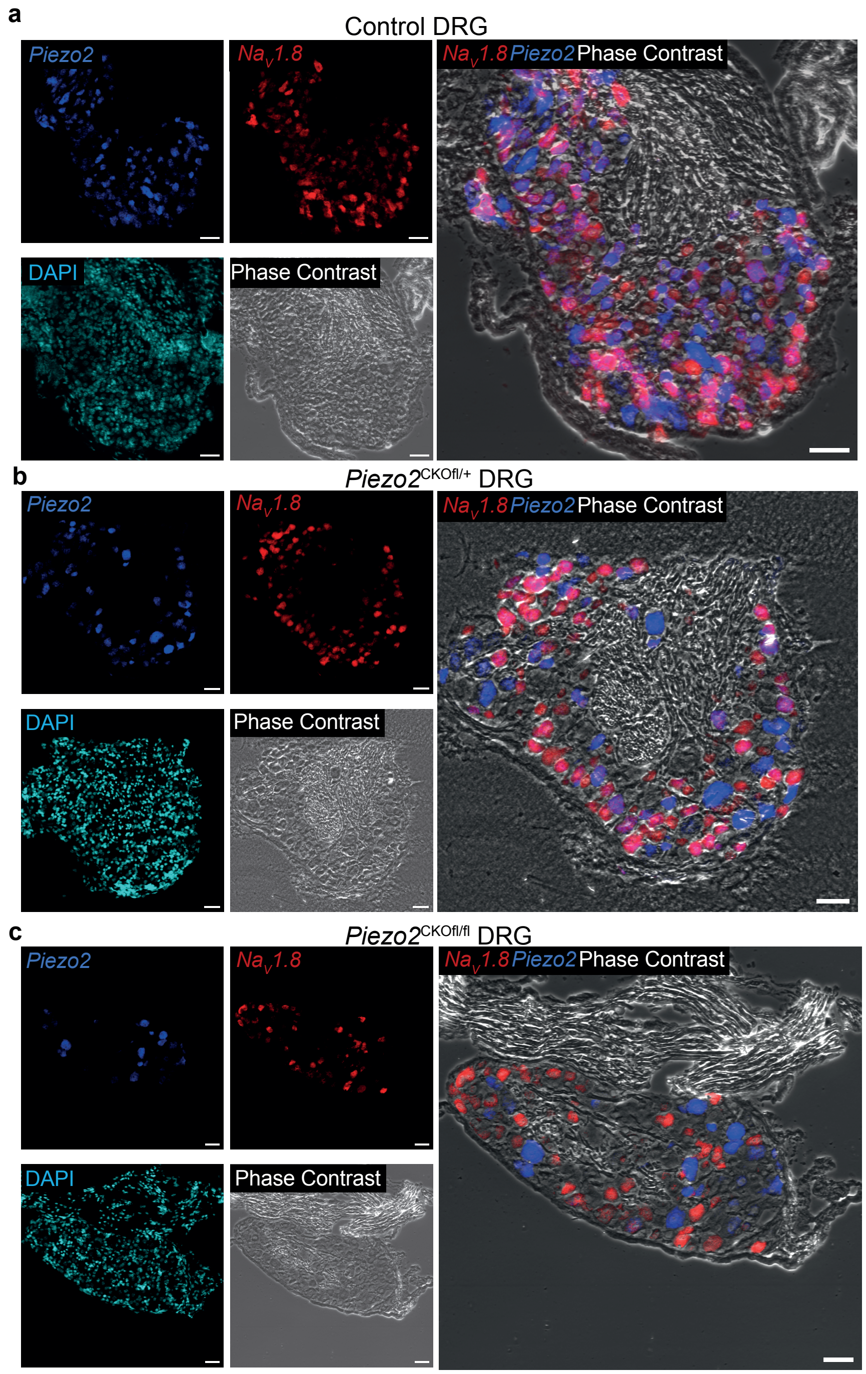

Supplemental Figure 3. Additional RNAscope pictures of mouse dorsal root ganglia (DRG) from same group of mice used in Figure 1. Pictures shown are examples from different mice than in Figure 1. **a** control, **b** *Piezo2*$^{\mathrm{CKOfl/+}}$ or **c** *Piezo2*$^{\mathrm{CKOfl/fl}}$ DRG with phase contrast and DAPI channels shown. Scale bar = 50 µm. N=4 no Cre controls; n=3 *Piezo2*$^{\mathrm{CKOfl/+}}$; n=5 *Piezo2*$^{\mathrm{CKOfl/fl}}$. For **a**, **b**, **c**, adjustments to individual colour channels on merged images was performed using brightness and contrast tools. Adjustments were made applied to the entire image and have been applied across all images and controls.

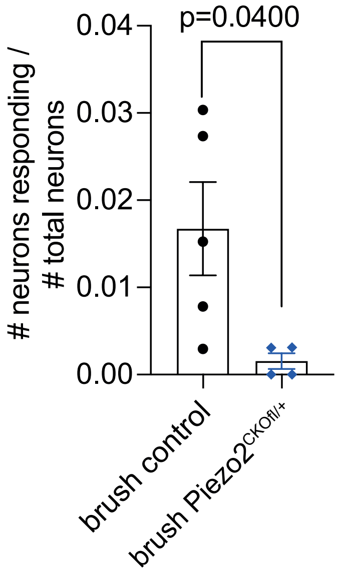

Supplemental Figure 4. Dynamic brush stimulus applied to the plantar surface of the hind paw. The number of Na_V1.8+ neurons responding was quantified and compared between strains by two-tailed unpaired t-test (each dot = one mouse; for each mouse >225 neurons imaged in total) (control, n=5 mice, black circles; *Piezo2*$^{CKOfl/+}$, n=4 mice, blue diamonds). Area of responding neurons: control: 28 neurons, 370±19 μm^2 (mean±SEM); *Piezo2*$^{CKOfl/+}$: 2 neurons, 267 μm^2; 330 μm^2. Source data are provided as a Source Data file.

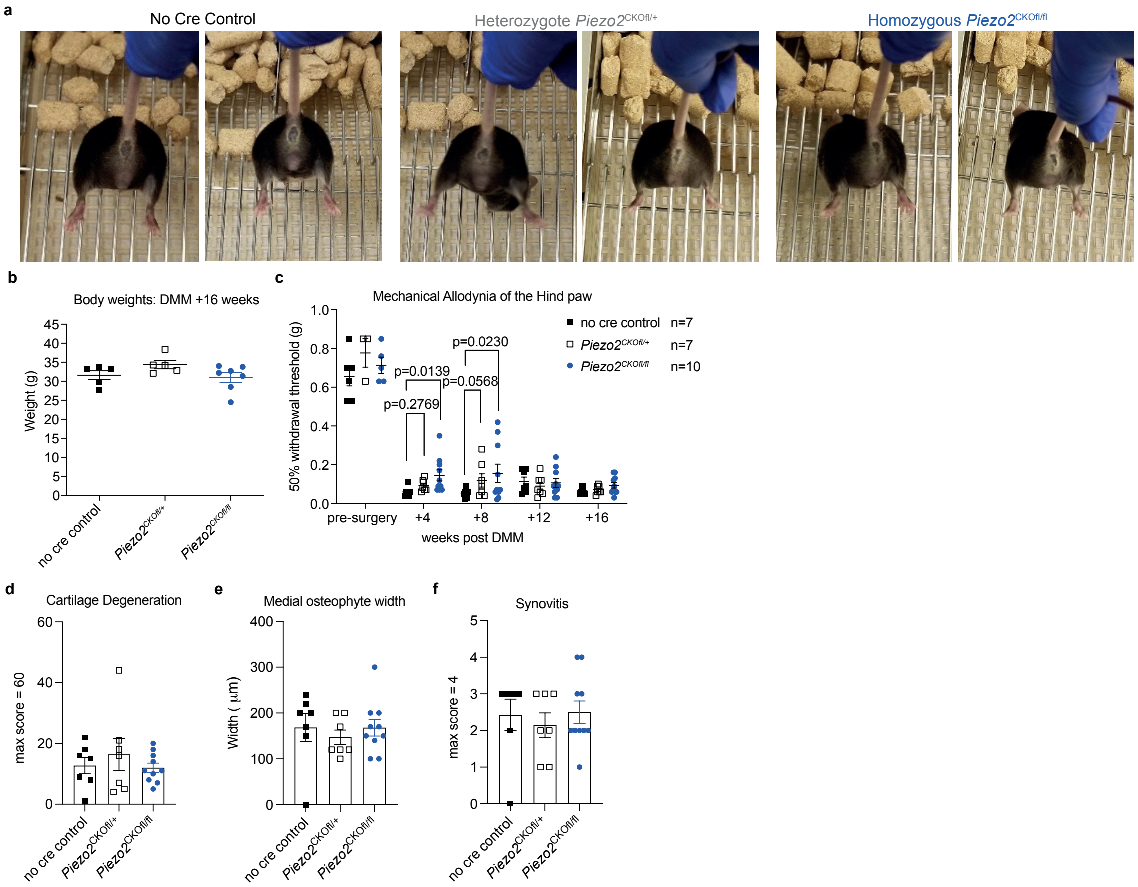

Supplemental Figure 5. An independent experiment showing that Piezo2 plays a role in mechanical sensitization in the destabilization of the medial meniscus (DMM) mouse model of osteoarthritis. **a** No effect on proprioception was detected with deletion of Piezo2 from nociceptors using Na$_V$1.8 cre. Two mice shown per genotype. **b** No change in body weight (g) was detected with deletion of Piezo2 from nociceptors (littermate no cre controls, n=5 mice, black squares), (*Piezo2*$^{CKOfl/+}$, n=5 mice, white squares), (*Piezo2*$^{CKOfl/fl}$, n=7 mice, blue circles). One-way ANOVA with Dunnett's multiple comparisons test, no cre vs. *Piezo2*$^{CKOfl/+}$ p=0.2544; no cre vs. *Piezo2*$^{CKOfl/fl}$ p=0.9226. **c** Hind paw mechanical allodynia was assessed on log-transformed data by two-way ANOVA with Sidak post-test (time 0 – only a subset of mice could be tested pre-surgery: (littermate no cre controls 'others', n=6), (heterozygous *Piezo2*$^{CKOfl/+}$, n=3), (homozygous *Piezo2*$^{CKOfl/fl}$, n=5)) (weeks 4-16: (littermate no cre controls 'others', n=7), (heterozygous *Piezo2*$^{CKOfl/+}$, n=7), (homozygous *Piezo2*$^{CKOfl/fl}$, n=10)). **d-f** represents modified OARSI scoring for **d** Total cartilage degeneration (medial + lateral compartments), assessed by one-way ANOVA with Sidak post-test (littermate no cre controls, n=7), (*Piezo2*$^{CKOfl/+}$, n=7), (*Piezo2*$^{CKOfl/fl}$, n=10). No cre vs. *Piezo2*$^{CKOfl/+}$: p=0.6925; no cre vs. Piezo2$^{CKOfl/fl}$: p=0.9838; **e** Osteophyte width, assessed by one-way ANOVA with Sidak post-test (littermate no cre controls, n=7), (*Piezo2*$^{CKOfl/+}$, n=7), (*Piezo2*$^{CKOfl/fl}$, n=10). No cre vs. *Piezo2*$^{CKOfl/+}$: p=0.7705; no cre vs. Piezo2$^{CKOfl/fl}$: p=0.9998; and **f** Synovitis, assessed by Kruskal-Wallis test with Dunn's post-test (littermate no cre controls, n=7), (*Piezo2*$^{CKOfl/+}$, n=7), (*Piezo2*$^{CKOfl/fl}$, n=10). No cre vs. *Piezo2*$^{CKOfl/+}$: p=0.7778; no cre vs. Piezo2$^{CKOfl/fl}$: p=>0.9999. Mean±SEM. 'Osteoarthritis Research Society International' = OARSI. For **b-f**, Source data are provided as a Source Data file.

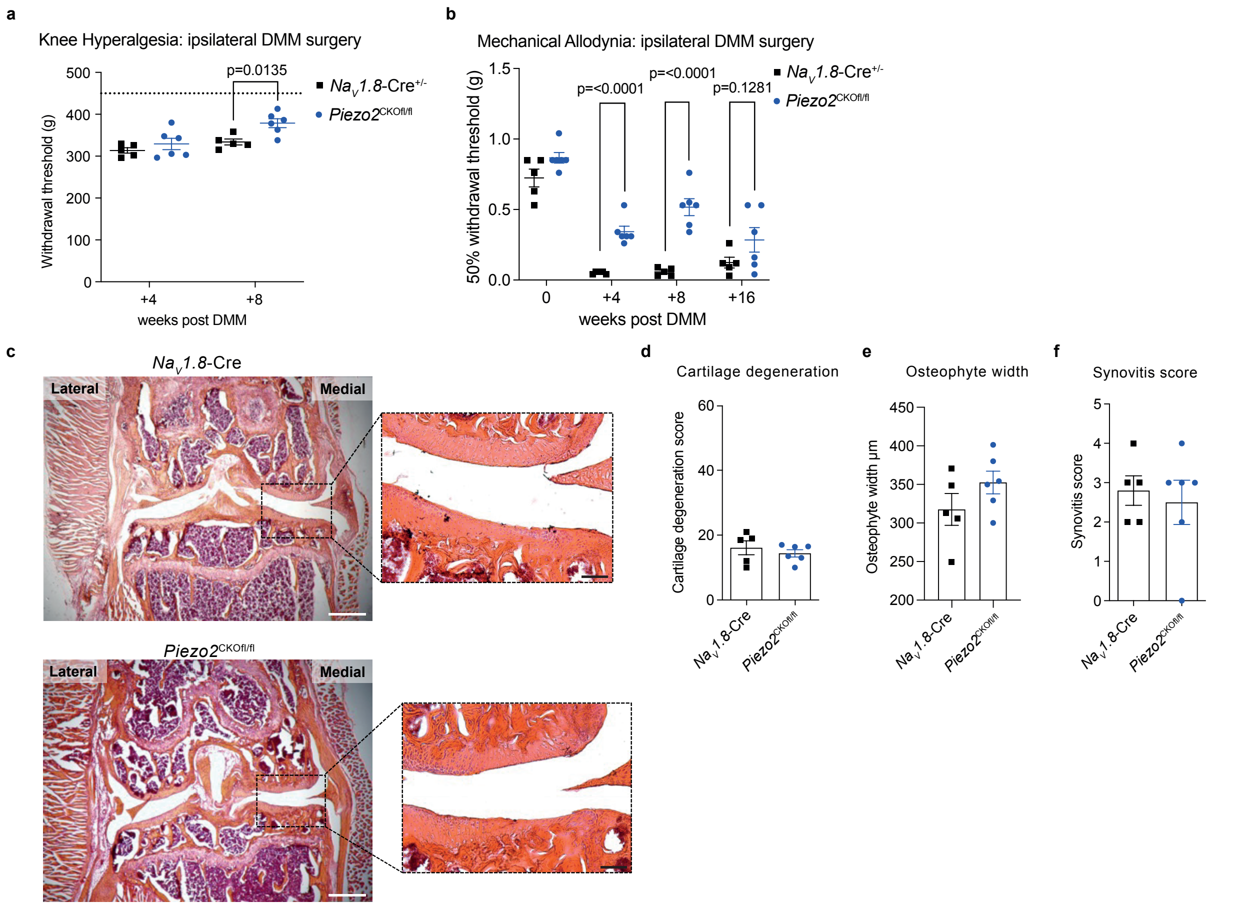

Supplemental Figure 6. A second independent experiment showing that Piezo2 plays a role in mechanical sensitization in the destabilization of the medial meniscus (DMM) mouse model of osteoarthritis. **a** Knee hyperalgesia was assessed in Na$_V$1.8 cre$^{+/-}$ (n=5, black squares) and *Piezo2*$^{CKOfl/fl}$ (n=6, blue circles) mice after DMM surgery. Dashed line indicates maximum of the assay: 450 g. Contralateral unoperated legs: 4 weeks (mean±SEM): Na$_V$1.8 cre$^{+/-}$ (444±4) and *Piezo2*$^{CKOfl/fl}$ (440±10). 8 weeks: Na$_V$1.8 cre$^{+/-}$ (443±4) and *Piezo2*$^{CKOfl/fl}$ (417±10). Two-way repeated measures ANOVA with Sidak post-test. **b** Hind paw mechanical allodynia was assessed in mice from part **a**. An independent experiment is shown in Supp. Fig. 5. Two-way repeated measures ANOVA with Sidak post-test. **c-f** Right knee joints of mice in **a,b** collected 18 weeks after DMM surgery and assessed for histology: **c** Representative histological images of the right knee joint. Scale bar for whole joint image on left = 500 µm. Scale bar for inset = 100 µm. **d-f** represents modified OARSI scoring (for the same mice in **a**) for **d** total cartilage degradation (medial + lateral compartments) (unpaired two-tailed t-test, p=0.4781), **e** osteophyte width (unpaired two-tailed t-test, p=0.1907); and **f** synovitis score (two-tailed Mann-Whitney test, p=0.9978), respectively. Mean±SEM. 'Osteoarthritis Research Society International' = OARSI. For **a,b,d-f**, source data are provided as a Source Data file.

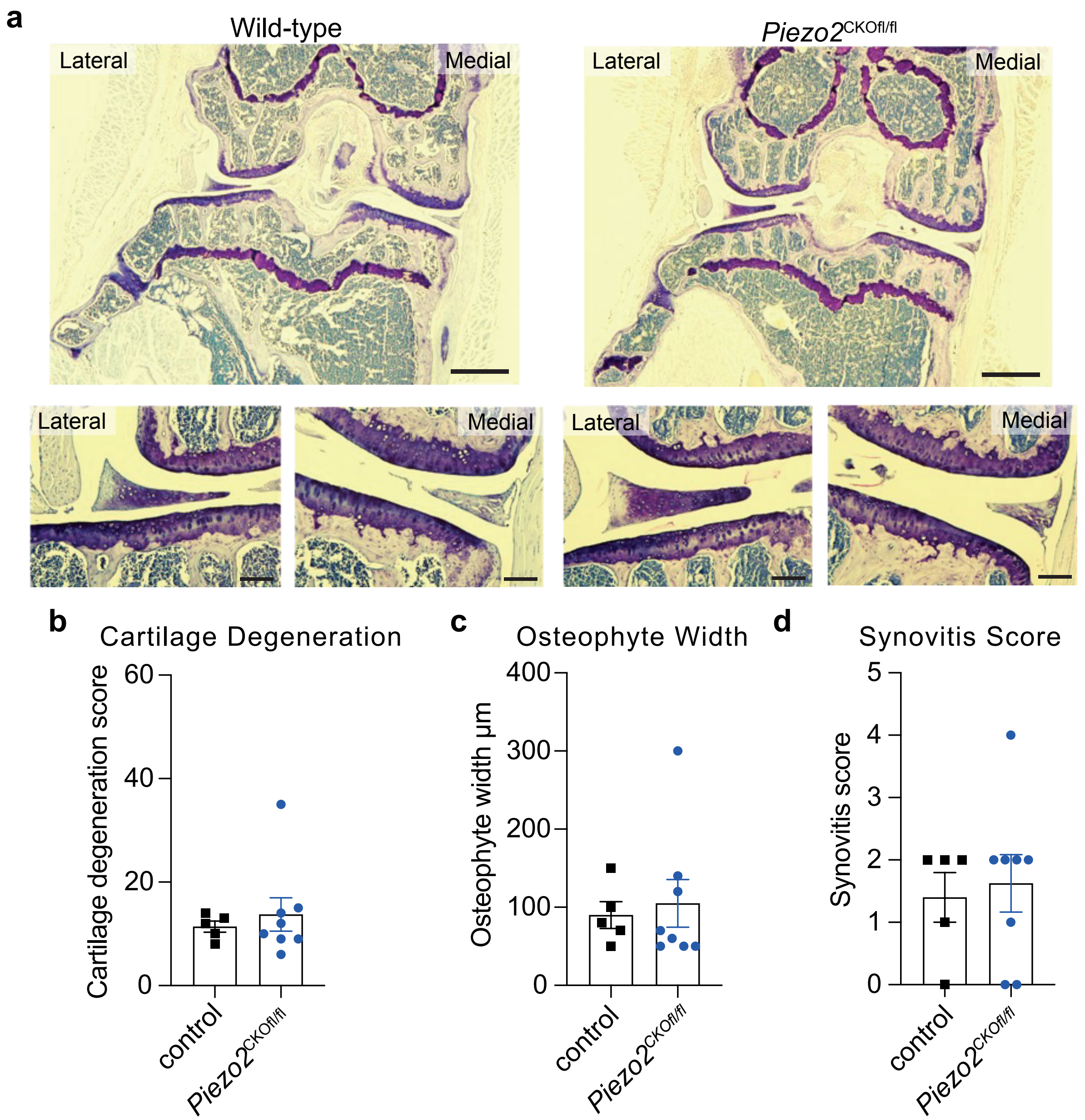

Supplemental Figure 7. Histology associated with spontaneous aging experiment in Figure 4C – right knee joints were collected when mice reached age 22 months. **a** Representative histological images of the right knee joint of littermate no cre controls (n=5, black squares) and *Piezo2*$^{CKOfl/fl}$ (n=8, blue circles) mice from the experiment corresponding to Figure 4C. Scale bar for whole joint image on top = 500 µm. Scale bar for inset = 100 µm. **b-d** represents modified OARSI scoring (for the same mice in **a**) for **b** total cartilage degradation (medial + lateral compartments), unpaired two-tailed t-test: no cre vs. *Piezo2*$^{CKOfl/fl}$: p=0.5875; **c** osteophyte width, unpaired two-tailed t-test: no cre vs. *Piezo2*$^{CKOfl/fl}$: p=0.7230; and **d** synovitis score, two-tailed Mann-Whitney test: no cre vs. *Piezo2*$^{CKOfl/fl}$: p=0.9239. Mean±SEM. For **b-d**, source data are provided as a Source Data file. 'Osteoarthritis Research Society International' = OARSI.

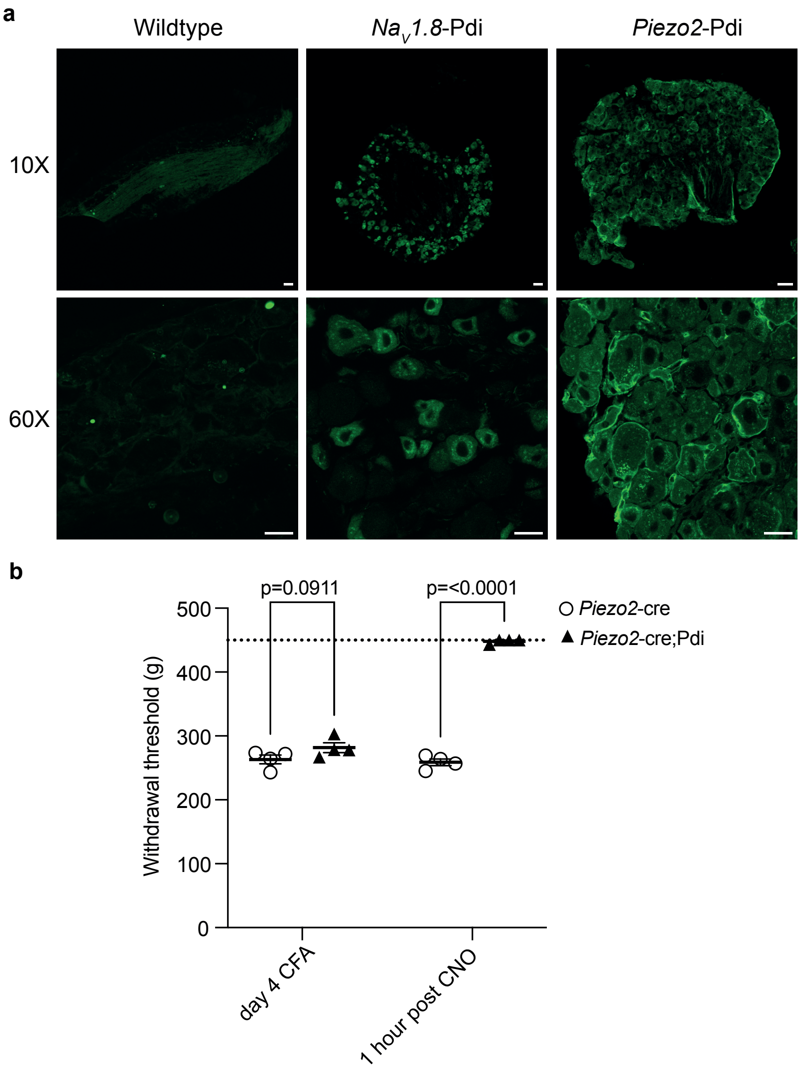

Supplemental Figure 8. Controls associated with Figure 5. **a** Mouse dorsal root ganglia (DRG) – additional hemagglutinin (HA)-tag staining images from Figure 5 at 10X and 60X magnification. Scale bar = 50µm (Top) and 25µm (Bottom). From left to right, images represent wildtype mouse (n=2, 12 weeks old), $Na_V1.8$-$Cre^{+/-}$;$Pdi^{fl/+}$ mouse (strain used in (*33*), used here as a positive control, n=1, 8 weeks old), and Piezo2-$Cre^{+/-}$;$Pdi^{fl/+}$ mouse (n=6, 22-31 weeks old). **b** Control experiment to test the effect of CNO in mice not expressing the Pdi inhibitory DREADD receptor: 12-week old male Piezo2-cre (white circles) or Piezo2-cre;Pdi (black triangles) mice were injected with CFA in the right knee as in the experiment shown in Figure 3. On day 4 after the injection, all mice had developed knee swelling (Piezo2-cre, 2.5±0.1 mm; Piezo2-cre;Pdi, 2.8 ±0.1 mm) and knee hyperalgesia ('day 4 CFA'). All mice were injected with 10 mg/kg CNO in the right knee as in Figure 5 and one hour later knee hyperalgesia was re-assessed. Piezo2-cre (n=4 mice/group) or Piezo2-cre;Pdi (n=4 mice/group). Piezo2-cre;Pdi mice had significantly reduced knee hyperalgesia following CNO injection compared to Piezo2-cre mice. Two-way repeated measures ANOVA with Sidak post-test. Dashed line indicates the maximum of the assay = 450 g. Mean±SEM. 'CFA' = Complete Freund's adjuvant; 'CNO' = Clozapine N-oxide; 'Designer Receptors Exclusively Activated by Designer Drugs' = DREADD.

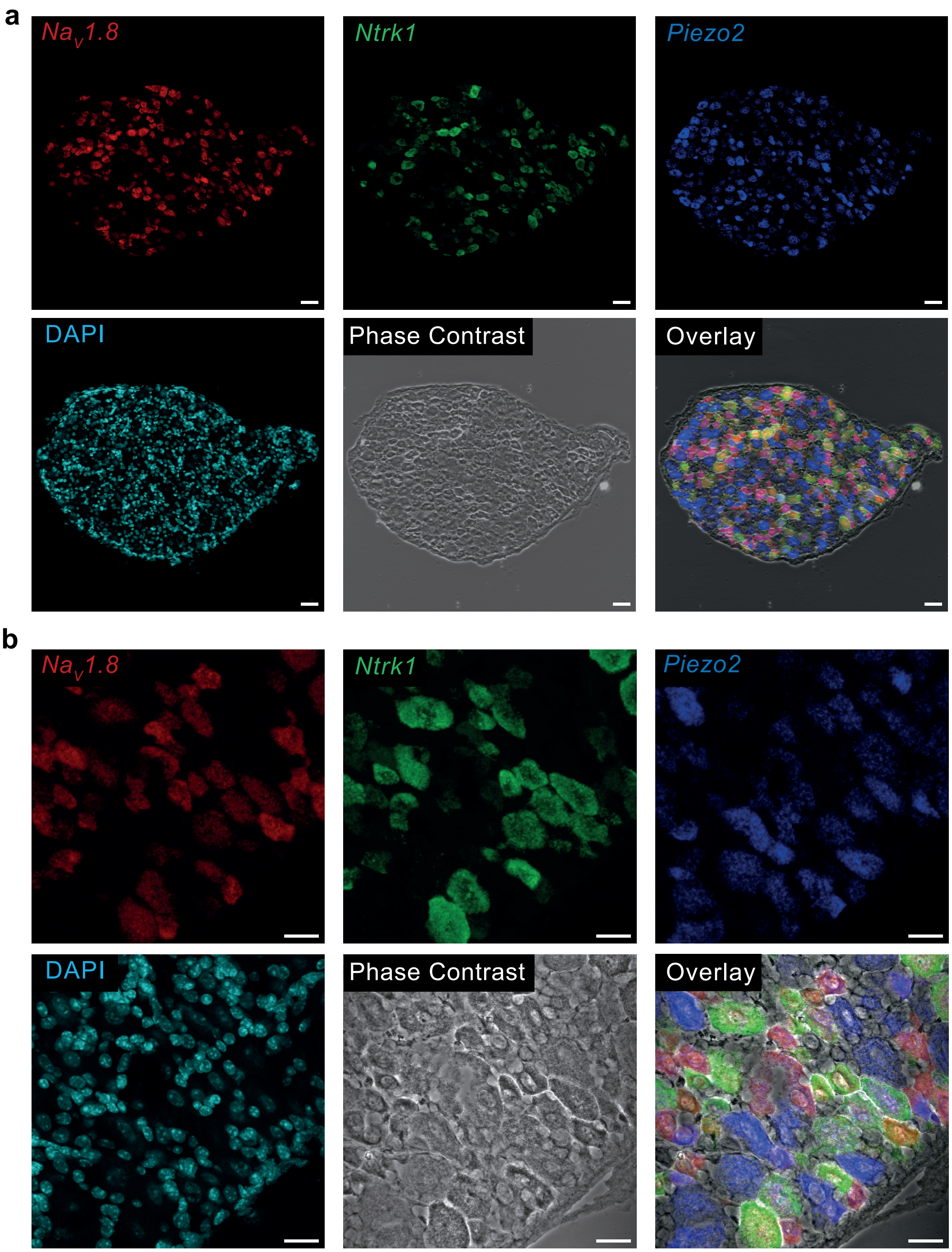

Supplemental Figure 9. Additional RNAscope pictures of mouse dorsal root ganglia (DRG) from same group of mice used in Figure 6. Pictures shown are examples of different mice than those shown in Figure 6. **a** 15X and **b** 60X magnification with phase contrast channel shown. Scale bar = 50 µm **a** and 25 µm **b**. Wild-type mouse DRG (n=3 mice). For **a**, **b**, adjustments to individual colour channels on merged images was performed using brightness and contrast tools. Adjustments were made applied to the entire image and have been applied across all images and controls.

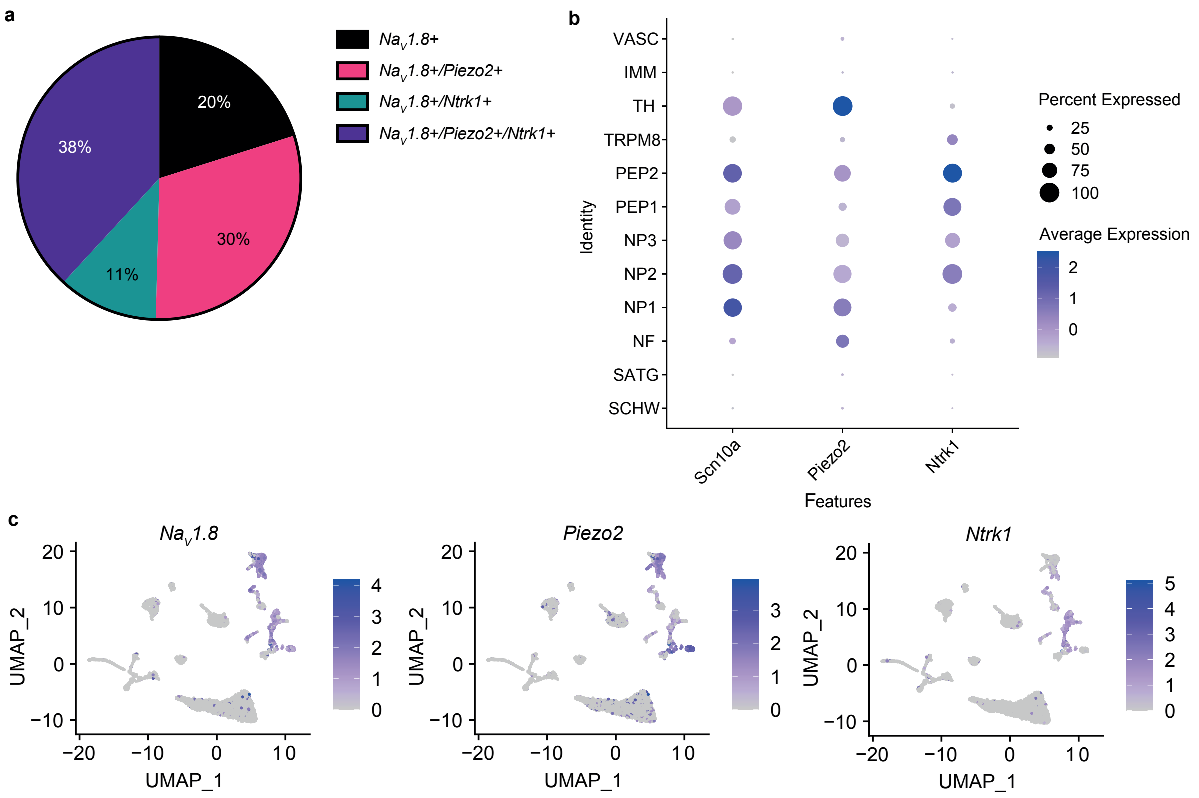

Supplemental Figure 10. Single cell RNAseq of mouse dorsal root ganglia (DRG) also demonstrates co-expression of *Na$_V$1.8*, *Piezo2* and *Ntrk1*. **a** Percentage of *Na$_V$1.8*+ neurons that are single positive, double positive with either *Piezo2* or *Ntrk1*, or triple positive (*Na$_V$1.8*+/*Piezo2*+/*Ntrk1*+). **b** Dot plot or **c** feature plots showing cluster distribution of these markers. 'SCHW' = Schwann cells; 'SATG' = satellite glia; 'NF' = neurofilament; 'NP' = non-peptidergic nociceptors; 'PEP' = peptidergic nociceptors; 'TRPM8' = transient receptor potential melastatin 8; 'TH' = tyrosine hydroxylase containing; 'IMM' = immune cells; 'VASC' = vascular cells.

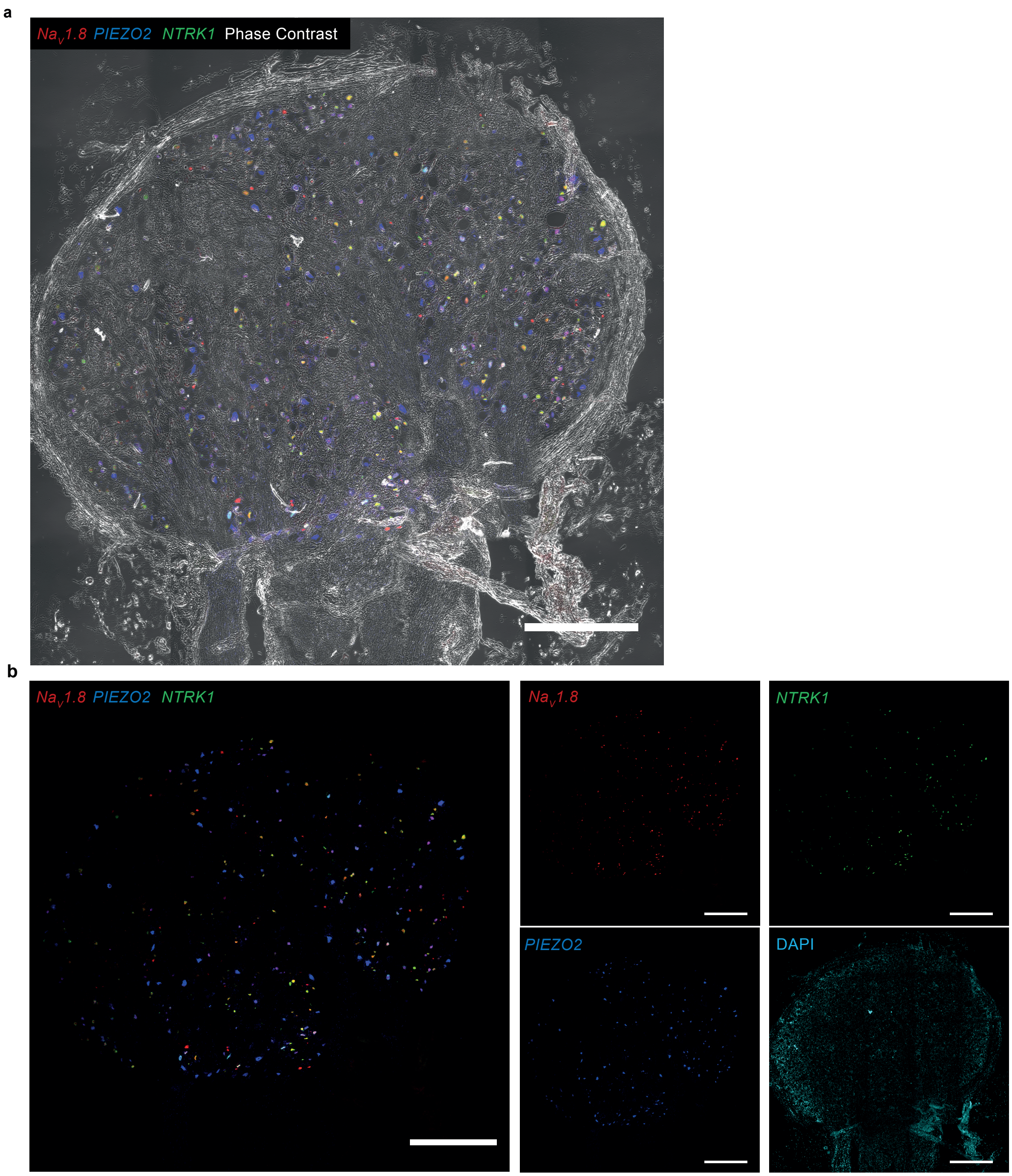

Supplemental Figure 11. Stitched image of male human dorsal root ganglion (DRG) – corresponding to Figure 6 showing phase contrast **a** and individual channels of *Na$_V$1.8, PIEZO2, NTRK1* and DAPI **b**. Scale bar = 1 mm. n=1 male donor (same donor as used in Figure 6). For **a**, **b**, adjustments to individual colour channels on merged images was performed using brightness and contrast tools. Adjustments were made applied to the entire image and have been applied across all images and controls.

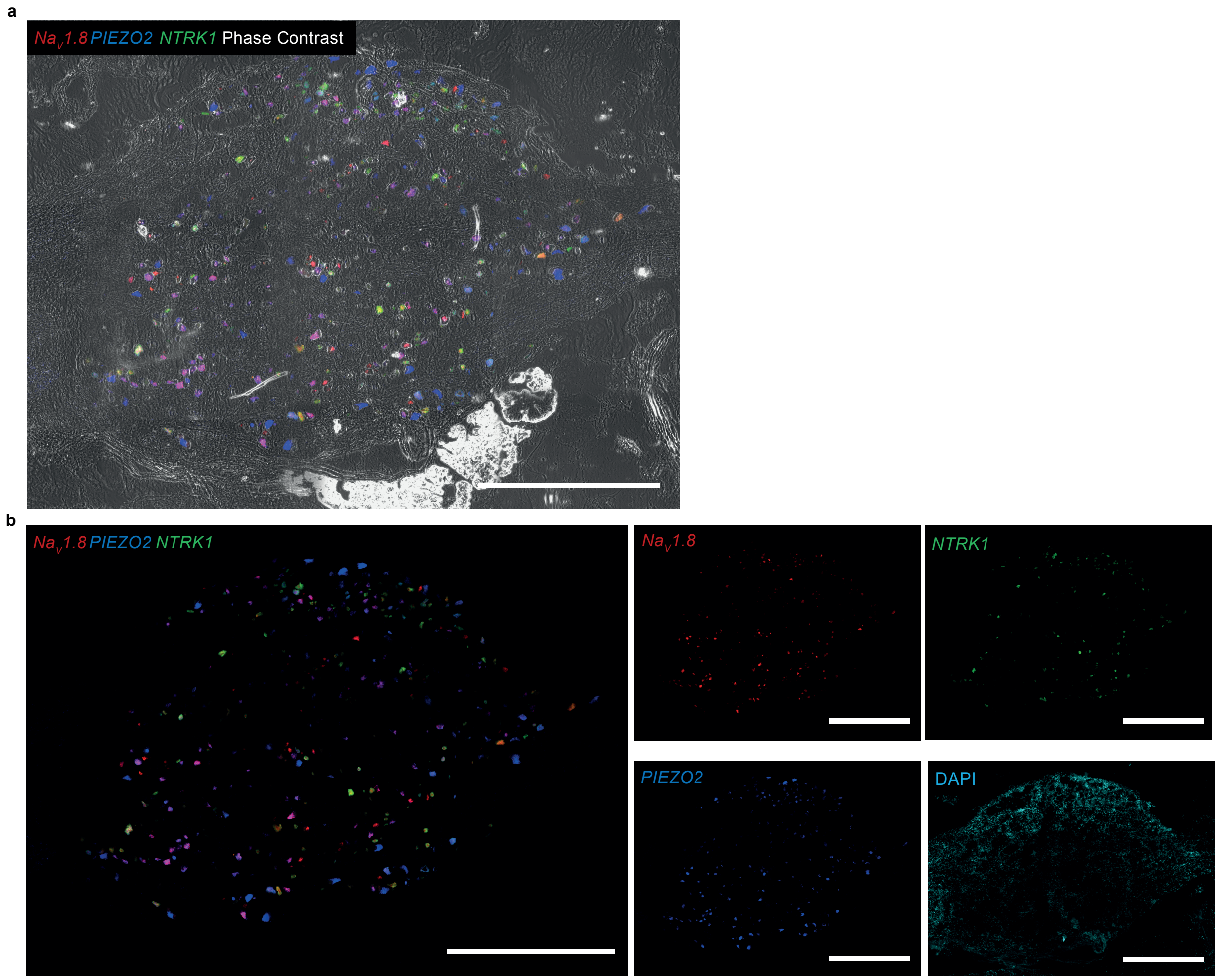

Supplemental Figure 12. Stitched image of female human dorsal root ganglion (DRG) – corresponding to Figure 6 showing phase contrast (A) and individual channels of *Na_V1.8, PIEZO2, NTRK1* and DAPI (B). Scale bar = 1 mm. Experiment repeated for n=2 female donors (same donors as shown in Figure 6). One sample displayed in **a**, **b**. For **a**, **b**, adjustments to individual colour channels on merged images was performed using brightness and contrast tools. Adjustments were made applied to the entire image and have been applied across all images and controls.

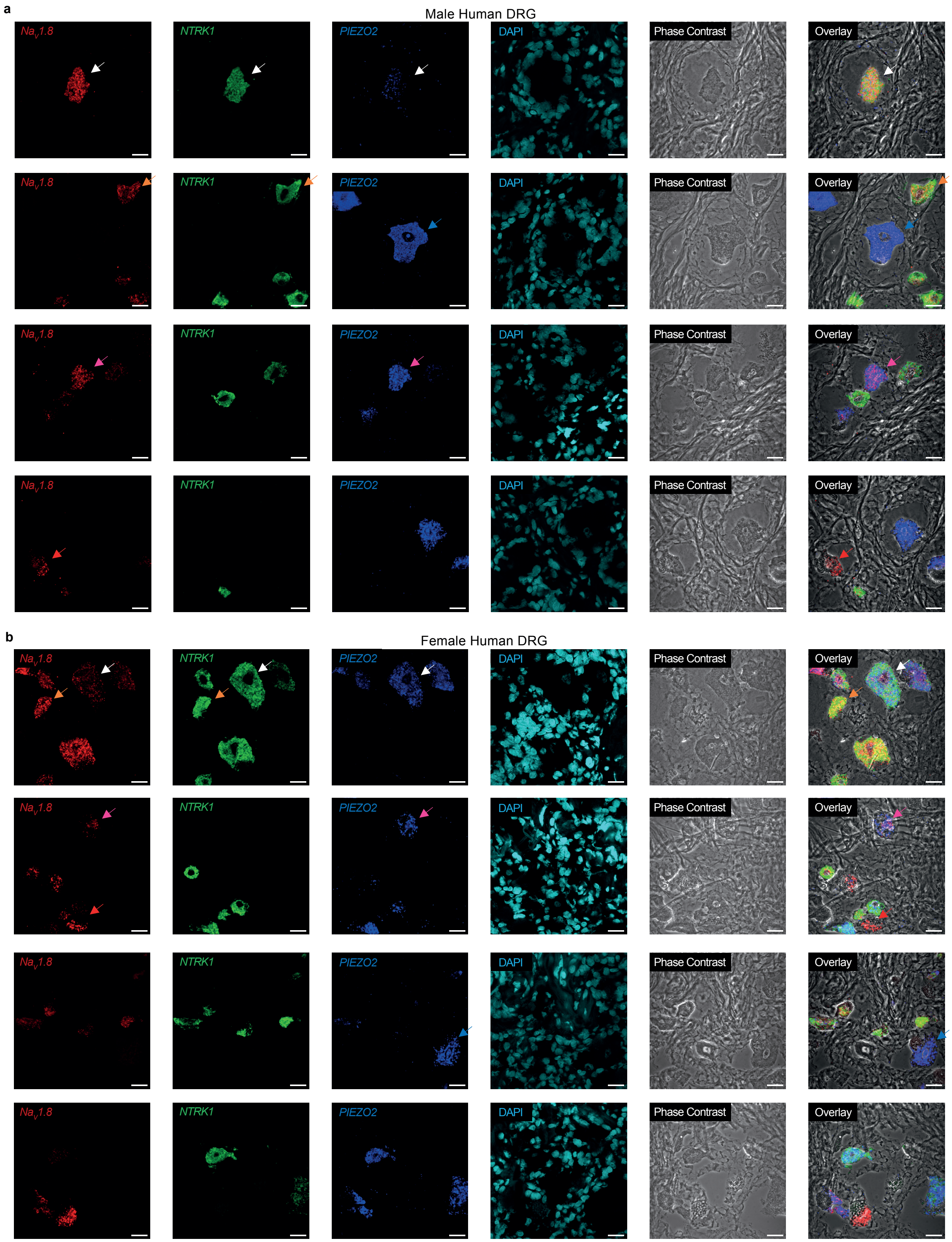

Supplemental Figure 13. Additional 60x images of human dorsal root ganglia (DRG) from Figure 6 to demonstrate different co-expression combinations of *Na$_V$1.8, NTRK1*, and *PIEZO2* in male (A)

and female (B) human DRG. White arrows indicate cells expressing *Na_V1.8, NTRK1*, and *PIEZO2.* Pink arrows indicate cells expressing *Na_V1.8* and *PIEZO2.* Orange arrows indicate cells expressing *Na_V1.8* and *NTRK1*. Red arrows indicate cells expressing only *Na_V1.8.* Blue arrows indicate cells expressing only *PIEZO2.* Scale bar = 25 µm. n=3 donors; two male and one female (same donors as shown in Figure 6). For **a**, **b**, adjustments to individual colour channels on merged images was performed using brightness and contrast tools. Adjustments were made applied to the entire image and have been applied across all images and controls.

Supplemental Code: text that can be used to create a Fiji macro to calculate Δ*F*/*Fo*:

```
//Video file that you wish to calculate Delta F/Fo on should be open in Fiji
run("32-bit");
x = getTitle();
Stack.getDimensions(width, height, channels, slices, frames);
rename("original");
//Change the number in the line below to reflect the number of frames you wish to use as Fo (baseline)
run("Z Project...", "stop=30 projection=[Average Intensity]");
selectWindow("original");
imageCalculator("Subtract create stack", "original","AVG_original");
close("original");
selectWindow("Result of original");
imageCalculator("Divide create stack", "Result of original","AVG_original");
close("AVG_original");
close("Result of original");
//Depending on the size of the video file and the computer speed it may take some time to calculate
//Wait for the window to pop up with the correct file name 'Result of Result of original'
selectWindow("Result of Result of original");
```
