# Supplementary material for: Piezo2 expressing nociceptors mediate mechanical sensitization in experimental osteoarthritis
Source: Nat Commun. 2023 Apr 29;14:2479. doi: 10.1038/s41467-023-38241-x (PMC10148822; doi:10.1038/s41467-023-38241-x)
Supplement: Supplementary file 3 — Description of Additional Supplementary Files [file 41467_2023_38241_MOESM3_ESM.pdf]

Description of Additional Supplementary Files

File Name: Supplementary Movie 1
Description: Video showing baseline, 30 g and 100 g responses of the control mouse DRG associated with Figure 2B.

File Name: Supplementary Movie 2
Description: Video showing baseline, 30 g and 100 g responses of the Piezo2$^{CKOfl/+}$ mouse DRG associated with Figure 2C.
