# Supplementary material for: Piezo2 expressing nociceptors mediate mechanical sensitization in experimental osteoarthritis
Source: Nat Commun. 2023 Apr 29;14:2479. doi: 10.1038/s41467-023-38241-x (PMC10148822; doi:10.1038/s41467-023-38241-x)
Supplement: Supplementary file 7 — Reporting Summary [file 41467_2023_38241_MOESM7_ESM.pdf]

nature portfolio

Corresponding author(s): Rachel E. Miller

Last updated by author(s): Apr 11, 2023

Reporting Summary

Nature Portfolio wishes to improve the reproducibility of the work that we publish. This form provides structure for consistency and transparency in reporting. For further information on Nature Portfolio policies, see our Editorial Policies and the Editorial Policy Checklist.

Statistics

For all statistical analyses, confirm that the following items are present in the figure legend, table legend, main text, or Methods section.

n/a	Confirmed	
☐	☒	The exact sample size (*n*) for each experimental group/condition, given as a discrete number and unit of measurement
☐	☒	A statement on whether measurements were taken from distinct samples or whether the same sample was measured repeatedly
☐	☒	The statistical test(s) used AND whether they are one- or two-sided<br>*Only common tests should be described solely by name; describe more complex techniques in the Methods section.*
☐	☒	A description of all covariates tested
☐	☒	A description of any assumptions or corrections, such as tests of normality and adjustment for multiple comparisons
☐	☒	A full description of the statistical parameters including central tendency (e.g. means) or other basic estimates (e.g. regression coefficient) AND variation (e.g. standard deviation) or associated estimates of uncertainty (e.g. confidence intervals)
☐	☒	For null hypothesis testing, the test statistic (e.g. *F*, *t*, *r*) with confidence intervals, effect sizes, degrees of freedom and *P* value noted<br>*Give P values as exact values whenever suitable.*
☒	☐	For Bayesian analysis, information on the choice of priors and Markov chain Monte Carlo settings
☒	☐	For hierarchical and complex designs, identification of the appropriate level for tests and full reporting of outcomes
☒	☐	Estimates of effect sizes (e.g. Cohen's *d*, Pearson's *r*), indicating how they were calculated

Our web collection on statistics for biologists contains articles on many of the points above.

Software and code

Policy information about availability of computer code

Data collection	PrairieView software version 5.3 or version 5.5; Olympus Fluoview FV10-ASW Ver.04.02
Data analysis	GraphPad Prism v9; Fiji 2.9.0; Cell Ranger v6.0.0; Seurat v4.0.1 R package; Custom ImageJ macro to calculate ΔF/Fo is included in the Supplementary Information file or can be downloaded here (https://mskpain.center/download_file/8dd82cc8-6c1c-4e37-b466-279afabd6307/530)

For manuscripts utilizing custom algorithms or software that are central to the research but not yet described in published literature, software must be made available to editors and reviewers. We strongly encourage code deposition in a community repository (e.g. GitHub). See the Nature Portfolio guidelines for submitting code & software for further information.

Data

Policy information about availability of data

All manuscripts must include a data availability statement. This statement should provide the following information, where applicable:

- Accession codes, unique identifiers, or web links for publicly available datasets
- A description of any restrictions on data availability
- For clinical datasets or third party data, please ensure that the statement adheres to our policy

Data Availability: Single cell RNAseq data have been deposited in NCBI GEO under accession number GSE198485 (https://www.ncbi.nlm.nih.gov/geo/query/acc.cgi?

acc=GSE198485). A reporting summary for this article is available as a Supplementary Information file. The source data underlying Figures and Supplementary Figures are provided in the Source Data file.

Human research participants

Policy information about studies involving human research participants and Sex and Gender in Research.

Reporting on sex and gender	Two dorsal root ganglia were from females and one was from a male
Population characteristics	ages 82-94, BMI, 21.2-27.6
Recruitment	Human DRGs were obtained post mortem from participants previously enrolled in the Religious Orders Study (ROS) or Rush Memory and Aging Project (MAP)
Ethics oversight	Human DRGs were collected post mortem from participants in the Religious Orders Study (ROS) or Rush Memory and Aging Project (MAP). At enrollment, participants agreed to annual clinical evaluation and organ donation at death, including brain, spinal cord, nerve, and muscle. Both studies were approved by the Institutional Review Board of Rush University Medical Center. All participants signed an informed consent, Anatomic Gift Act, and a repository consent to allow their resources to be shared.

Note that full information on the approval of the study protocol must also be provided in the manuscript.

Field-specific reporting

Please select the one below that is the best fit for your research. If you are not sure, read the appropriate sections before making your selection.

☒ Life sciences ☐ Behavioural & social sciences ☐ Ecological, evolutionary & environmental sciences

For a reference copy of the document with all sections, see nature.com/documents/nr-reporting-summary-flat.pdf

Life sciences study design

All studies must disclose on these points even when the disclosure is negative.

Sample size	Sample sizes were chosen based on previous studies utilizing these outcome measures (PMID: 23185004, 33827672, 28380690, 28992367, 29563338, 31351964)
Data exclusions	No data exclusions.
Replication	All attempts at replication were successful. Independent replicate numbers varied by experiment but in general were performed at least 3 times.
Randomization	Cages of mice were randomly allocated to treatment group.
Blinding	For behavioral assessments, investigators were blinded to group allocation during data collection/analysis. For histological scoring, investigators were blinded while scoring. For other experiments, it was not possible to blind while performing the data collection and all data analysis was performed similarly across groups.

Reporting for specific materials, systems and methods

We require information from authors about some types of materials, experimental systems and methods used in many studies. Here, indicate whether each material, system or method listed is relevant to your study. If you are not sure if a list item applies to your research, read the appropriate section before selecting a response.

Materials & experimental systems

n/a	Involved in the study	
☐	☒	Antibodies
☒	☐	Eukaryotic cell lines
☒	☐	Palaeontology and archaeology
☐	☒	Animals and other organisms
☒	☐	Clinical data
☒	☐	Dual use research of concern

Methods

n/a	Involved in the study	
☒	☐	ChIP-seq
☒	☐	Flow cytometry
☒	☐	MRI-based neuroimaging

Antibodies

Antibodies used	Alexa Fluor 488 conjugated anti-HA.11 Epitope Tag antibody (Biolegend catalog #: 901509)
Validation	We performed testing as follows: Negative control: absence of staining in tissue not expressing the HA-tag. Positive control: replication of staining in tissue known to express the HA-tag and previously published (PMID: 28380690). According to the manufacturer's website, each lot of this antibody is quality control tested by immunocytochemistry. The HA.11 antibody recognizes the influenza hemagglutinin epitope (YPYDVPDYA) which has been used extensively as a general epitope tag in expression vectors. The extreme specificity of the antibody allows unambiguous identification and quantitative analysis of the tagged protein. The HA.11 antibody recognizes HA epitopes located in the middle of protein sequences as well as at the N- or C-terminus.

Animals and other research organisms

Policy information about studies involving animals; ARRIVE guidelines recommended for reporting animal research, and Sex and Gender in Research

Laboratory animals	Species: Mice;<br>Strains: NaV1.8-Cre; tdTomato loxp (Jax # 007909); GCaMP6s loxp (Jax # 028866); Piezo2-Cre (Jax # 027719); Piezo2 loxp (Jax # 027720); hM4Di-loxp; C57BL/6J; All mice used were on a C57BL/6 background.<br>Ages: up to 22 months of age - detailed information can be found for each experiment in the methods section
Wild animals	The study did not involve wild animals.
Reporting on sex	Human DRGs: male and female<br>CFA experiment: female mice<br>DMM, aging, NGF experiments: male mice<br>Mouse DRGs: male and female mice<br>Detailed information can be found in the methods section.
Field-collected samples	The study did not involve samples collected from the field.
Ethics oversight	All animal experiments were approved by the Institutional Animal Care and Use Committees at Rush University Medical Center and Northwestern University.

Note that full information on the approval of the study protocol must also be provided in the manuscript.
